# A case of severe megacolon due to acquired isolated hypoganglionosis after low anterior resection for lower rectal cancer

**DOI:** 10.1007/s12328-019-01079-2

**Published:** 2019-12-11

**Authors:** Tetsuro Tominaga, Satoshi Nagayama, Manabu Takamatsu, Shun Miyanari, Toshiya Nagasaki, Tomohiro Yamaguchi, Takashi Akiyoshi, Tsuyoshi Konishi, Yoshiya Fujimoto, Yosuke Fukunaga, Masashi Ueno

**Affiliations:** 1 Department of Gastroenterological Surgery, Gastroenterological Cancer, Cancer Institute Hospital, Japanese Foundation for Cancer Research, 3-8-31 Ariake, Koto-ku, Tokyo, 135-8550 Japan; 2 Division of Pathology, The Cancer Institute, Japanese Foundation for Cancer Research, Tokyo, Japan

**Keywords:** Acquired isolated hypoganglionosis, Megacolon, Laparoscopic total colectomy

## Abstract

Acquired isolated hypoganglionosis is a rare intestinal neurological disease, which presents in adulthood with the clinical symptoms of chronic constipation. A 39-year-old man underwent laparoscopic low anterior resection and covering ileostomy for locally advanced-rectal cancer. A 6-month course of postoperative adjuvant chemotherapy was completed, followed by closure of the ileostoma. After the closure, he developed severe colitis which required 1-month of hospitalization. Mucosal erosions and pseudo-membrane formation were evident on colonoscopy and severe mucosal damage characterized by infiltration of inflammatory cells and crypt degeneration were pathologically confirmed. Even after the remission of the colitis, he suffered from severe constipation and distention. At 4 years after the stoma closure, he decided to undergo laparoscopic total colectomy. Histopathologically, the nerve fibers and ganglion cells became gradually scarcer from the non-dilated to dilated regions. Immunohistochemical staining examination confirmed that the ganglion cells gradually decreased and became degenerated from the normal to dilated region, thereby arriving at the final diagnosis of isolated hypoganglionosis. The patient recovered without any complications and there has been no evidence of any relapse of the symptoms. We present a case of acquired isolated hypoganglionosis-related megacolon, which required laparoscopic total colectomy, due to severe enterocolitis following stoma closure.

## Introduction

Isolated hypoganglionosis is a hypogenetic variant of intestinal dysganglionosis characterized by the decrease and degeneration of ganglion cells in the lamina propria in the region of the colon and rectum [1]. This rare disease represents about 3–5% of all intestinal neurological diseases, which presents with the clinical symptoms of chronic constipation and pseudo-obstruction [2]. The disease has been classified into two subtypes: congenital IHG (CIHG) and acquired IHG (AIHG) [3]. In a nationwide survey over 10 years in Japan, only eight patients were identified as having AIHG, whereas 104 patients had CIHG [4]. Because of its low incidence, the definitive concept, etiology, and histopathological criteria of AIHG remain controversial [5, 6]. Regarding the treatment of AIHG, if conservative treatment is not effective for improving the symptoms, surgery may be a promising treatment option [7]. We encountered an AIHG patient who developed marked megacolon after severe enterocolitis following the closure of a covering ileostoma and treated him successfully by laparoscopic total colectomy.

## Case report

**Fig. 1:**
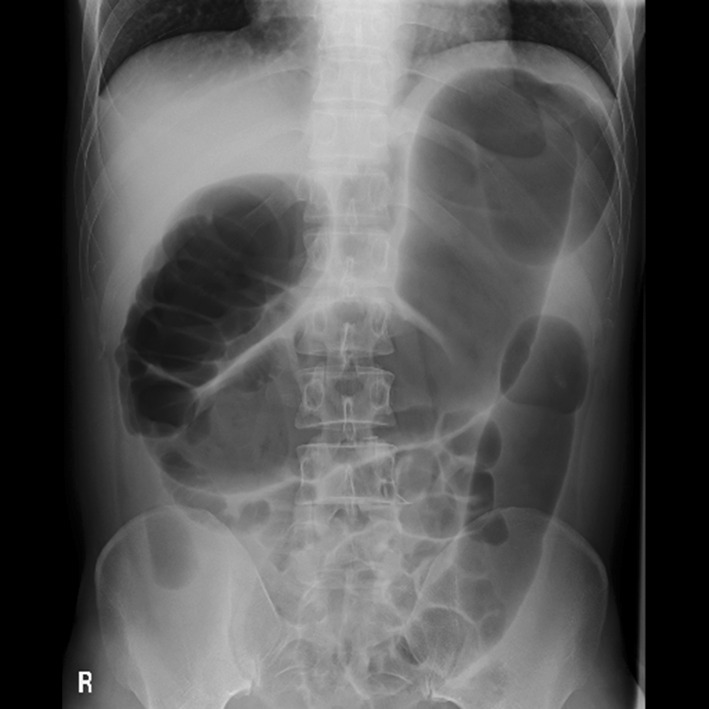
Abdominal X-ray after stoma closure. Large intestine was markedly dilatated

**Fig. 2:**
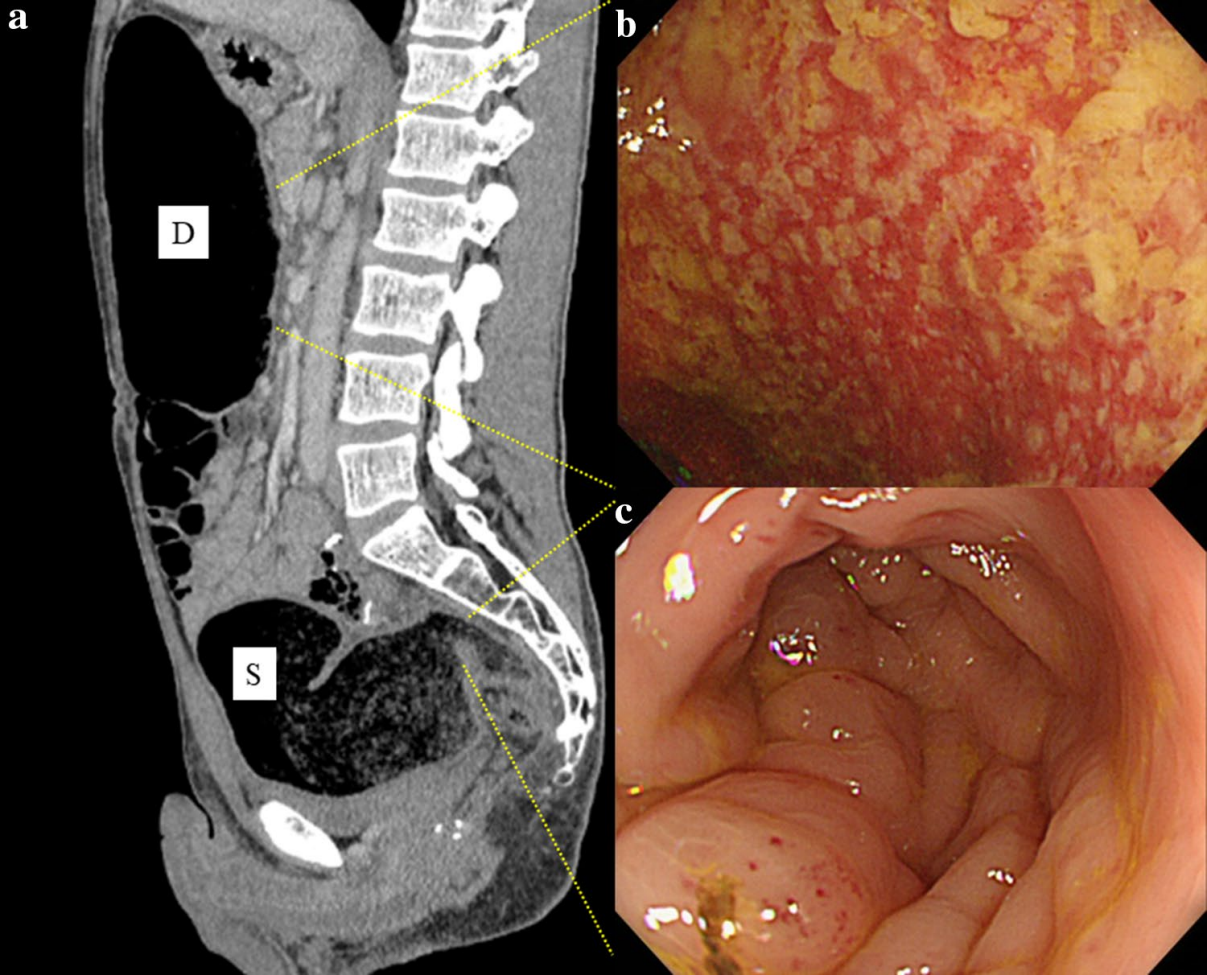
Abdominal CT and colonoscopy findings following stoma closure. CT showed a marked dilatation from the ascending to sigmoid colon (**a**). Colonoscopy revealed a mucosal erosion and pseudo-membrane in the descending colon (**b**). Colonoscopy also revealed an edematous mucosa in the rectosigmoid colon (**c**). Pseudomembranous enterocolitis or cytomegalovirus-induced enterocolitis were suspected

**Fig. 3:**
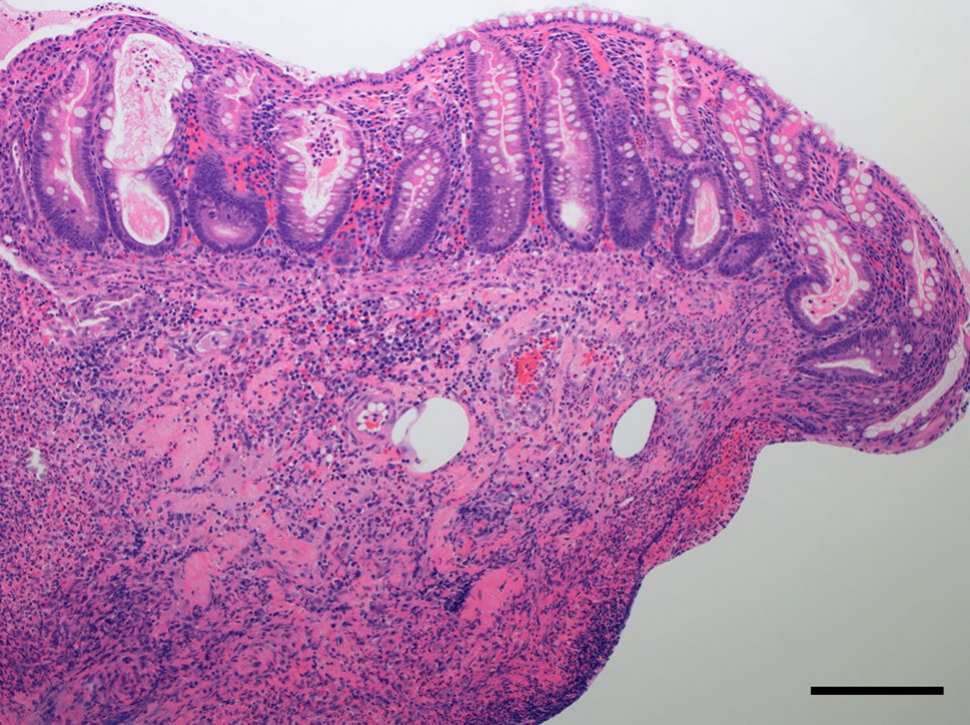
Microscopic findings of the biopsied specimen at the time of severe colitis in the descending colon (hematoxylin and eosin staining). There was extensive inflammatory cell infiltration and a substantial number of degenerated crypts, resulting in marked erosive changes in the colon. Scale bar indicates 200 µm

**Fig. 4:**
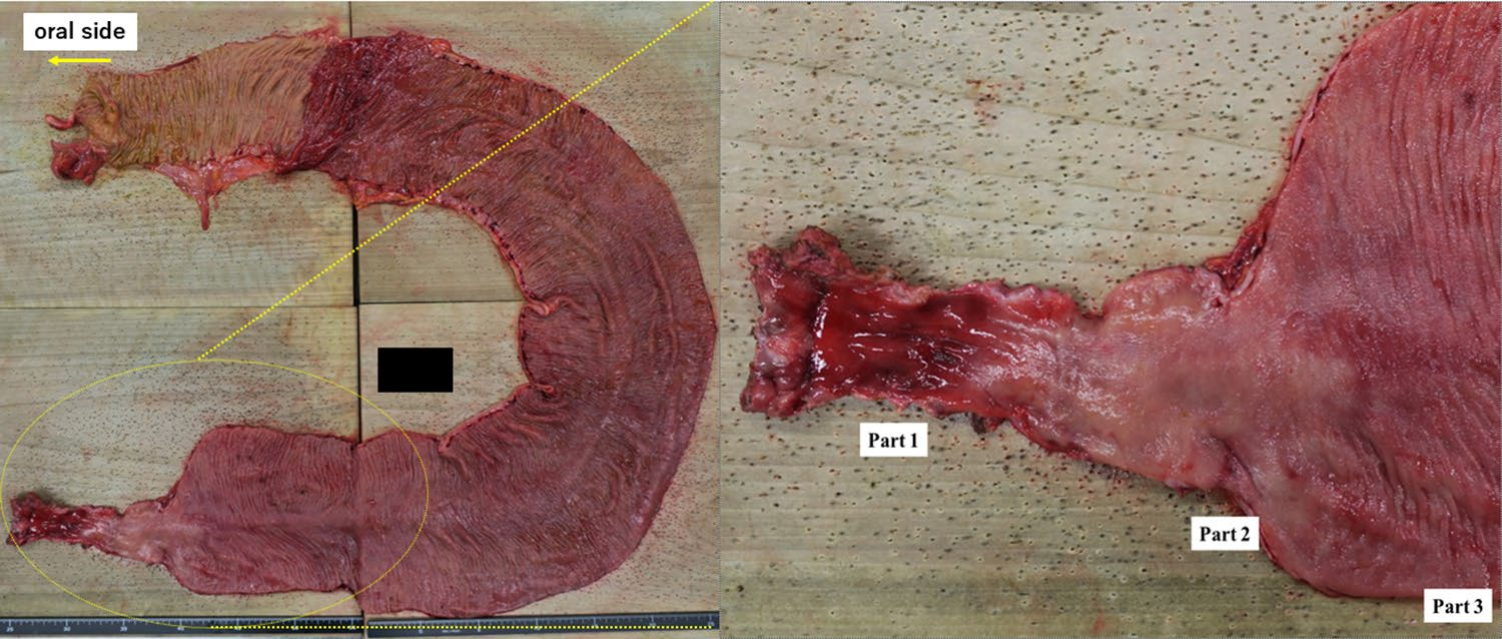
Macroscopic findings of the resected specimen. Note a marked dilatation from the ascending to sigmoid colon

**Fig. 5:**
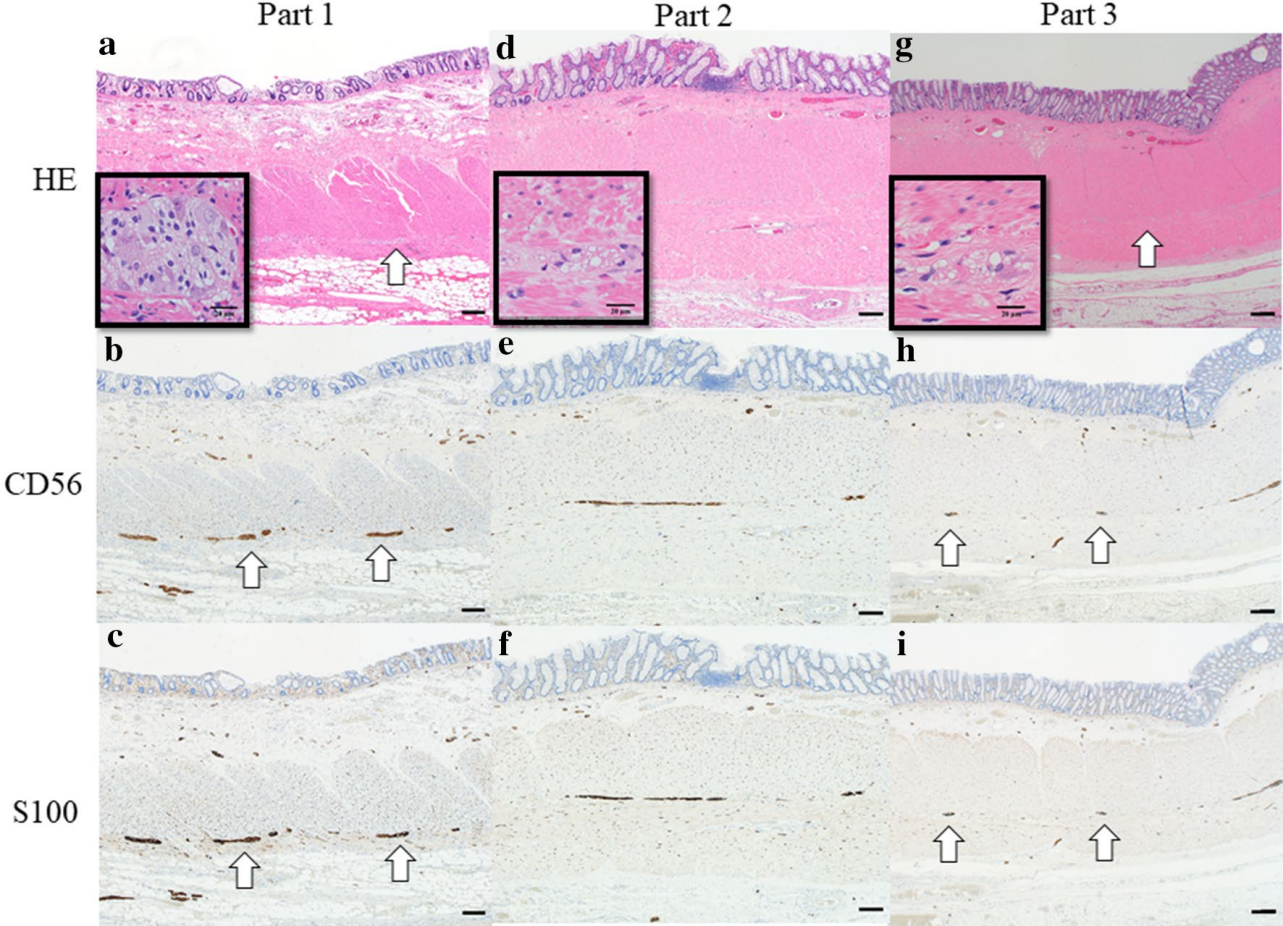
Microscopic findings of the resected specimen. The number of nerve fibers and ganglion cells were decreased in the dilated area (*g*, arrow) but unchanged in the non-dilated area (**a**, arrow). Immunohistochemistry for CD56 and S-100 showed conspicuous neural components (**b**, **c**, **h**, and **i**). Note the differences in density of neural fibers and ganglion cells (arrow). Scale bar indicates 200 µm

A 39-year-old man was diagnosed with locally advanced lower rectal cancer. He had no family history of bowel disease. He received preoperative chemoradiotherapy (50.4 Gy) followed by laparoscopic low anterior resection (LAR) and covering ileostomy. His postoperative course was uneventful. The final stage was determined as pT2pN1aM0 (p-Stage IIIB) after pathological examination. Adjuvant chemotherapy with XELOX over a 6month period was completed without severe adverse effects. At 7 months after LAR, the covering ileostoma was closed. On the fourth postoperative day, he developed abdominal distention with high fever and severe abdominal pain. Abdominal X-ray and CT showed remarkable bowel dilatation of the entire colon (Fig. 1), along with increased inflammation status (WBC 10,300/µL, CRP 10.3 mg/L). Colonoscopy revealed mucosal erosions and pseudo-membrane formation suspected of being pseudomembranous or cytomegalovirus-induced enterocolitis (Fig. 2). Although colonic mucosal erosion and severe inflammatory cell infiltration with crypt abscesses were evident on the biopsied samples, there were no findings detected suggestive of *Clostridium difficile* or cytomegalovirus infection (Fig. 3). Furthermore, there were no specific findings in the biopsied samples from the non-dilated area. Since the culture tests of the stool and blood samples were negative for any specific pathogens, he was diagnosed with severe enterocolitis of an unknown cause after stoma closure. Empirical treatment by intravenous administration of broad-range antibiotics and fasting was required for 1 month until the symptoms subsided. Even after the remission of the colitis, he suffered from severe constipation and abdominal distention. Abdominal CT scans showed wall thickening of the entire colon during the period of the severe colitis. In addition, follow-up CT examination revealed a remarkable bowel dilatation and stool retention throughout the colon at 2 months after the stoma closure. In spite of the persistent constipation, he chose for his condition to be monitored closely without any interventions. At 4 years after the stoma closure, he decided to undergo laparoscopic total colectomy to alleviate the symptoms. A trans-anal ileus tube was indwelled to improve the dilatation of the colon, which enabled us to accomplish laparoscopic total colectomy with a total operation time of 457 min and blood loss of 200 ml. The patient recovered gradually without any complications. Macroscopic findings showed marked dilatation and wall thickening of the entire colon without any mucosal lesions (Fig. 4). Histopathological examination of the resected specimen revealed that the nerve fibers and ganglion cells were intact in non-dilated area and they gradually decreased from non-dilated to dilated regions (Fig. 5a–i). In addition, some ganglion cells were degenerated in the dilated region. Immunohistochemical analysis of CD56 and S-100 showed a significant decrease in the number of neural cells in the Auerbach’s plexus.

## Discussion

Hirschsprung’s disease is a widely recognized condition characterized by congenital absence of intestinal ganglia in the plexus. On the other hand, a few adults who present with the clinical symptoms of chronic constipation, pseudo-obstruction, and acute abdomen are diagnosed with an adult onset of AIHG [8, 9]. A nationwide survey over 10 years in Japan showed that only 8 (7.1%) of 112 patients with isolated hypoganglionosis were diagnosed with AIHG [3]. A challenging issue is that there is a lack of definitive diagnostic criteria and consensus on effective treatment because of the rarity of AIHG [3].

AIHG has been histopathologically characterized by decreased ganglion cells, degeneration of ganglion cells, and ganglionosis in Auerbach’s plexus [10]. More recently, a reliable method for establishing a histopathological diagnosis of AIHG has been proposed using immunohistochemical examination of S-100, Hu C/D and CD56 [11]. S-100 and Hu C/D are used as neuronal markers in both peripheral and central nerves, which facilitate the identification of individual neurons [12–14]. CD56, known as NCAM1 (Neural cell adhesion molecule 1), is a multifunctional glycoprotein belonging to the immunoglobulin superfamily, involved in synaptic plasticity, neurogenesis, and the development of neural circuitry [15]. In the present study, pathological and immunohistochemical evaluation including S-100 and CD56 showed a decrease in the number of and degeneration of the ganglion cells in the dilatated area (Fig. 5). Taking these pathological findings and the clinical course into consideration, we speculated that hypoganglionosis in the colon might result in substantial hypoperistalsis, leading to the chronic retention of the stool and subsequent bowel dilatation.

According to a previous report on AIHG, the patients developed the symptoms later in life with a long period of chronic constipation or intestinal pseudo-obstruction [16]. In the present case, the patient had neither past history of bowel disease nor related family history. The abdominal distention first occurred after the severe colitis. The pathological findings confirmed that this case was not congenital but acquired IHG. Congenital hypoganglionosis is characterized by impaired craniocaudal migration of neuroblasts [3]. On the other hand, AIHG has been reported to be associated with Chagas disease [5], viral infection [17], ischemia [18], and infiltration of eosinophils [11]. The infectious agents might cause a severe damage to ganglion cells, resulting in their degeneration and death [3]. However, because of its rarity, there has been no consensus on the specific cause of this disease [19–21]. One common event in all cases is that the intestine was severely damaged by pathogens or disorders prior to the development of the disease. In this case, stool tests at the time of severe colitis were negative for *Clostridium difficile* and cytomegalovirus. There was no evidence of specific bacteria or virus infection on the biopsied specimens.

Since the adjuvant chemotherapy was administered after the primary surgery in this case, the ileostoma closure was performed 7 months after the first surgery. According to a retrospective analysis on stoma closure by Rubio-Perez and colleagues, postoperative complications increased significantly when ileostoma closure was delayed [22]. In 93 patients who underwent stoma closure, the average delay of closure was 10.3 months. The most frequent postoperative complications were ileus (13%), wound infection (13%), and pseudomembranous colitis (4%). The severe colitis might result from bacterial overgrowth, the presence of harmful agents, nutritional deficiencies, and disturbance in the relationship between mucosal layer and luminal bacteria [23]. Neut and colleagues reported that there was an increase of nitrate-reducing bacteria in patients with diversion colitis [24]. Since nitrate-reducing bacteria could produce nitric oxide which is toxic to colonic tissue, high concentration of nitric oxide was supposed to play a role in the development of the diversion colitis [25]. Recently, other factors including ischemia and immune disorder were considered to cause severe colitis after stoma closure [26, 27]. A further research is needed to clarify the mechanisms underlying the diversion colitis.

With respect to cancer treatment including chemotherapy and radiotherapy, a recent study has reported that over 80% of patients treated with 5-fluorouracil developed gastrointestinal mucositis [28]. It is well documented that both radiotherapy and chemotherapy including 5-fluorouracil or oxaliplatin could cause gastrointestinal toxicities such as direct epithelial injury, tissue ischemia, villi shortening, increased crypt depth, apoptosis of intestinal epithelial cells, and bacterial translocation [28–33]. In addition, cancer treatment might have a marked effect on gut microbiota, leading to intestinal dysbiosis, mucositis, and bacterial translocation [34]. In this case, the patient received preoperative chemoradiotherapy (oral 5-fluorouracil plus 50.4 Gy) and oxaliplatin-based adjuvant chemotherapy for 6 months. Although each of these factors could cause significant histological changes in the nerve fibers and ganglion cells independently, we speculated that a complex combination of the adverse effects of these treatments, including chemotherapy and radiotherapy, and the delay of the ileostoma closure could contribute to the development of severe colitis and subsequent AIHG.

As for the treatment of adult hypoganglionosis, surgery is the definitive treatment, when conservative treatment cannot alleviate the symptoms effectively [7]. Some patients with AIHG have developed necrosis or volvulus of the bowel that required emergency open-surgery [21, 35]. In most AIHG cases, however, the conditions deteriorate quite slowly, and therefore elective surgery can be considered. Currently, laparoscopic total colectomy can be performed more safely than open surgery [36]. Furthermore, laparoscopic surgery can reduce postoperative small bowel obstruction due to fewer adhesions, which is thought to be more beneficial for relatively younger patients with AIHG. In the present case, a trans-anal ileus tube was indwelled for 2 weeks preoperatively so that the markedly dilated colon was sufficiently deflated, thereby facilitating laparoscopic total colectomy without any intraoperative problems.

We encountered a case of AIHG due to severe colitis after stoma closure. Laparoscopic total colectomy was performed safely after preoperative intestinal decompression using a trans-anal ileus tube, and the patient was able to return to ordinary daily life.
